# Assistive Methodologies for Parkinson's Disease Tremor Management—A Health Opinion

**DOI:** 10.3389/fpubh.2022.850805

**Published:** 2022-04-26

**Authors:** V. Dineshkumar, D. Raveena Judie Dolly, D. J. Jagannath, J. Dinesh Peter

**Affiliations:** ^1^Department of Electronics and Communication Engineering, Karunya Institute of Technology and Sciences, Coimbatore, India; ^2^Department of Computer Science and Engineering, Karunya Institute of Technology and Sciences, Coimbatore, India

**Keywords:** Parkinson's disease, assistive devices, tremor, postural instability, non-invasive

## Introduction

Parkinson's disease makes lives challenging every day due to the evolving and progressive motor symptoms such as tremors, slow movements, postural instability, and stiffness. These physical symptoms can then in turn affect the thoughts, leading to a state of depression. Tremor is an involuntary, unintended, periodic movement of the muscle of one or more parts of the body and can affect the head, legs, or arms, but predominantly affects the hands. This paper provides an overview on controlling the Parkinson's tremor in the hand through assistive methodologies. Non-invasive low cost assistive devices are considered to reduce the hand tremor caused by Parkinson's disease. Artificial Intelligence tools offer insights to evaluate speech disorders of Parkinson's patients. It can also identify them based on facial expressions.

## Parkinson's—in the Perspective of Health

Parkinson's is a neurodegenerative disorder (1, 6, 15) that occurs due to the death of dopaminergic neurons (15). Electrophysiology is a way to examine the patients in the way of past events and physical exams. Our paper aims to provide a detailed survey on Parkinson's tremors and the ways that they can be detected (1), controlled (3, 13, 17), and analyzed (5, 17). The assessment of Parkinson's disease is based on the clinical interview, the physical examination, and structured instruments (15). Drawbacks to the use of clinical ratings include the reliance on real-time human vision to quantify small differences in motion and significant inter-rater variability due to inherent subjectivity in scoring the procedures. Tremor is an involuntary, unintended, periodic movement of the muscle of one or more parts of the body that can affect the head, legs, arms, and predominantly the hands. Parkinson's tremor can be detected with the help of the active particular muscles in the hand at the time of movement (8, 22). To detect the tremor, the subject should sit in a comfortable place and rest their hands (1). The hand tremor in Parkinson's disease is a periodic signal which has a frequency range (1–3, 6) where the number of oscillation/per time can be noted. The frequency can be calculated manually in the time domain by the total number of cycles/per second. But it is not an easy task in the case of random signals with many frequencies. To study the tremor in the human body, knowledge about the natural frequency is very important. Based on the physical properties, each part of the body will oscillate based on its frequency. The oscillation is like a mechanical (1, 5, 17) component for tremors detected a change in their frequency when the mass is loaded. The energy comes from the irregular rate of the motor units. Tremor can be produced in one or more structures in the CNS; it causes an oscillation and transmits with the motor system. At that point, the tremor is at the origin, with no change in frequency, but the mass is present. A comparison of frequency in the limbs with several oscillations in the generation of tremors is considered. If a single oscillation is produced, all the limbs generate the same frequency. If the frequency is varied, there may be independent oscillators. Those oscillators play an important role in diagnosis. The frequency range of the Parkinson's tremor is between 4 and 7 Hz as a “rest tremor” (1). To detect hand tremors, accelerometer (1–3, 9, 15, 20), Gyrosocope (1, 4, 10, 17), EMG (8, 22) techniques, and IMU (13, 16, 17) motion sesnors are used.

## Assistive Devices—for Health Enhancement

Numerous devices have been proposed to improve lives in the medical arena. Certain devices are designed to help clinicians with accurate diagnosis. Certain other devices provide support to the patients. Devices with low cost may be useful for people on lower incomes. This paper highlights an assistive devices for Parkinson's disease patients experiencing tremor in the hands.

### Tuned Vibration Absorber

Hashem et al. (2), proposed a tuned vibration absorber is proposed to suppress the vibrations in the human arm experimental model with two degrees of freedom. From the perspective of dynamics, the degree of freedom is actuated by more than two muscles. The DOF model consists of two pairs of springs to replace the muscles. The aim of this technique is to reduce the tremor, with the help of the human arm model theoretically and numerically; one pair of elbow muscles and the other parallel to the shoulder muscle is considered. In the vibration control approach, a spring-mass damper with an oscillatory system is taken to extract or absorb the vibrations. The vibration absorber is made up of another combination of mass-spring dampers which is added to reduce the amplitude of the vibration. The PD tremor has a frequency range of around 2–12 Hz. It involves a broadband vibration control issue; to eliminate this, they implement the vibration absorber. To create a physical prototype, TVA has proof mass, tuning structure, and body. The proof/absorb mass is used to dissipate the vibrational energy. The tuning structure consists of a beam spring and guild slider. The body with the case is attached to the forearm. The frequency response of the two joints determines the correlation in controlled and uncontrolled cases.

### Intelligent Glove

Kazi et al. (3) designed an intelligent glove and a piezoelectric actuator are proposed to control unintentional trembling. The IV Training Arm Tremor model (2) is intended to collect the data to induce vibration in the human forearm. The rig holds the hand model in a horizontal axis to match the postural tremor. The unbalanced masses with DC motors (2) are used for the exciting source to enhance the postural tremor behavior. The assessment of the rig and real human hand tremor can be recorded and measured. Accelerometer (1) and laser displacement sensor are used to measure the displacement and acceleration of the hand tremor. The amplitude of vibration can be described in terms of acceleration and displacement in the time and frequency domain, resulting in the suppression of the glove (12, 14, 18). The frequency (2) of acceleration signal, displacement signal (2), and piezoelectric frequency gives an excellent way to reduce the tremor. The advantage of this technique is the glove with IV training arm tremor model which will be able to reduce the tremor.

### Signal Sensors

Deep Brain stimulation is a surgical procedure for Parkinson's disease. There has been no accurate monitoring system using this simulation effect till now. A sensor module was devised by Dai and D'Angelo (4) that combines the accelerometer (1, 3, 15) and gyroscope (1) using MEMS technology. The sensor module is placed at the tip of the finger and sends the measured data to the computer through USB. The assessment is done with the help of sensor data and some adaptive algorithms to categorize their severity level with a standard rating scale through linear regression model and lists the scale (UPDRS) values (15, 21) in Graphical User Interface. This method is considered to be more advantageous as it is purely non-invasive. By processing, a spectral analysis and statistical analysis on the sensor data for tremor quantification has been performed.

### Adaptive Tremor Cancellation

Pathak et al. (5) suggested reducing the tremor and stabilizing the hand by active cancellation technology. The advantage of this technique is that the device is compact, non-invasive, and lightweight. The ACT consists of a power supply unit, sensor, and motion generating platform. An accelerometer (1, 15) is integrated into the spoon to measure the direction of the spoon in x and y directions. The two DC motors (2, 3) are connected with mechanical burdens and coupled with a spoon in both directions. The peak amplitude of displacement (2) can be analyzed by the signal extraction method.

### Cantilever Vibration Control

Srivani Padma et al. (6) proposed a measurement device based on the cantilever vibration method. Due to tremors, strain is varied and this variation creates vibrations on the cantilever and is attained in the Fiber Bragg Grating sensor to measure the vibration from a hand tremor. This device is placed on the backhand and the patient holds it. Because of strain variation, the vibration data can be recorded with the help of a sensor, and FFT is applied to the recorded data to get a frequency response (2, 3). The FBG sensor has a fast response, low fatigue, and self-regard from electromagnetic interference which is considered to be advantageous. This device is used for entry-level diagnostic purposes.

### Wearable Motion Tracker

Papini et al. (7) proposed a programmable hand tremor concept to suppress the hand tremor through simulation. This tool is based on the wrist-haptic interface with comparable space, and the wrist is attached to the novel end effector spherical joint. The user's wrist exerts the controlled forces, while the frequency (2, 3) and amplitude can be correlated with the people having tremors.This method proves to be ideal since it is non-invasive. Using an optical motion tracking system, a tracking-based algorithm has been developed, with the help of a tremor signal from the patient. The user stands in front of the table (2) and the device is attached to the user's forearm to explore the objects. This device can be controlled by comparing with trajectories of the tremor-affected people. In this device, they produced a low-frequency motion from the users and focus to improve the functionality of the system.

### Adaptive Control System

Xu et al. (8) designed a closed-loop system to examine the tremor from Parkinson's disease. This closed-loop system consists of a DSP module, front module, and TENS module. In the front module, surface EMG (1, 22) applies to the flexor digitorum superficial muscles and amplifies. Conversion of analog into digital through sampling and analysis of the tremor signal using the DSP module and TENS module is discussed. The closed-loop system can be operated in two modes: auto mode and testing mode. The testing mode is used to modify the parameters manually such as amplitude, frequency, and pulse width for the TENS. In auto mode, the DSP module processes the sampling of the surface EMG (22) and detects the presence of tremor through spectrum analysis. If a tremor happens, the DSP gives the trigger signal to TENS to simulate the pulses from the testing mode parameters. To detect tremors due to Parkinson's disease, biphasic current pulses of EMG signal are utilized to suppress the tremors on the lateral surface of the hand based on a predefined threshold, which is considered to be the highlight of the proposed method. Experiments were performed on healthy and tremor patients to validate this closed-loop system.

### Wearable Wrist Watch

Jeon et al. (9) proposed a low-power, wearable device that is that measures the tremor signal from Parkinson's patients using a wristwatch type device that consists of a triaxial gyroscope (1) that has (+/-) 2,000 dps and a triaxial accelerometer (1, 3, 15) which has (+/-) 16 g along all the axes. The measurements adopted were displacement (2, 3, 5) and acceleration at the same time for tremor analysis. The device is placed in the wrist and middle fingers of both hands. If a severe tremor symptom occurs, the device could be placed on other fingers. The signals can be recorded when the patient is at a rest position and video is also recorded for further evaluation.

### Sensors Activated Wearable Glove

Turkistani et al. (10) proposed a glove (3) with inbuilt vibrator sensors that produce oscillations and minimize hand tremors. The glove also consists of accelerometer (1, 15)-gyroscope (1, 4)-based MEMS (4) motors that can be placed on each finger and interface with the microcontroller which is an easy way to connect the vibration motors and output pins of the Arduino board. The data represents the vibration changes in the x,y,z directions. The vibration motors play a major role to reduce the tremors in the fingers. In this device, the reduction of the tremor in up to 40%.

### IoT Interfaced Control

Sachindra Ragul et al. (11) developed an IoT-based device to control and perform frequency analysis of the hand tremors. A flex sensor and an accelerometer (1, 15) are placed in the glove (3, 10) and it is used to measure the amplitude and frequency of the hand tremors. The control module consists of a microcontroller (10) with a Wifi module that is used to upload the tremor data to the cloud and is monitored by the doctors. The Wifi communication can be improved and maintained by particle photons. Most tremors happen in the hand, so by placing a highspeed rotating disc on the hand, the hand tremor is stabilized. The methodology seems to be of very low cost and low power compared with the EMG technique (8, 22).

### Role of Artificial Intelligence

Artificial Intelligence offers a way to many medical miracles. It assists humans in every possible way in the present era. AI is a boon to mankind and will serve as a platform for many assistive devices in the field of medicine. To analyze a Parkinson's patient based on facial expressions, speech disorder is a field of research that would definitely provide a new way of life and hope to people. Diagnosis and treatment with the help of AI is developing quickly in the medical field.

## Discussion

**Figure 1:**
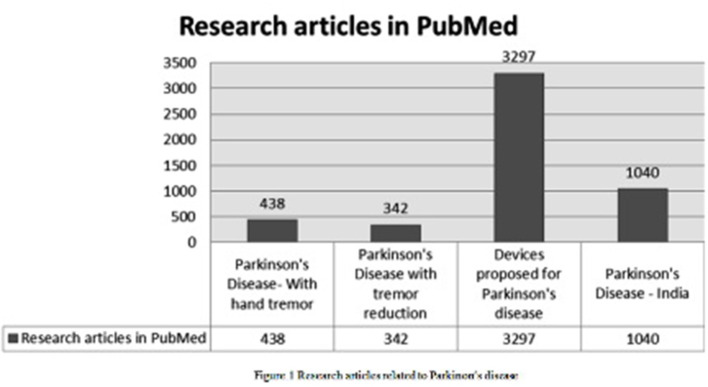
Research articles related to Parkinson's disease.

Human beings face many challenging diseases day to day. Research and technology has become vital to identifying these diseases and diagnosign, curing, and alleviating health conditions. Parkinson's disease make lives difficult every day due to the progressing motor symptoms such as tremor, slow movements, imbalance, and stiffness. These symptoms in turn affect the thoughts, leading to a state of depression. The survey indicates that many research proposals and devices are evolving to manage the effects that occur due to Parkinson's disease. The research articles to diagnose and cure PD from 1957 proposed under various categories out of 79,766 articles are depicted in Figure 1.

This indicates that research has been continually in process for early diagnosis and proper treatment of PD.

Tremor is an involuntary, unintended, periodic movement of the muscle of one or more parts of the body which can affect the head, legs, or arms, but predominantly the hands. The tremor (12, 19) caused due to PD begins in a limb and progresses toward the hand and fingers. This is generally termed as a pill—rolling tremor where the movement rubs the thumb and forefinger back-and-forth. It occurs when the hand is at rest. Risk factors include age, heredity, exposure to toxins, and gender.

The vibrations caused due to tremors of Parkinson's disease patients are hoped to be managed and stabilized. The hand movements can be assisted with the help of a device. The assistive device needs to be very comfortable to wear, portable, lightweight, and stabilize the hand when tremors occur. With the assistive device, the patient having tremors can perform their activities without any external support.

## Author Contributions

VD and DD devised the work, the main conceptual ideas, the proof outline, and worked out almost all of the technical details. DJ and JP worked on the manuscript. All authors contributed to the article and approved the submitted version.

## Funding

This article was based on the work supported and funded by the Indian Council of Medical Research (ICMR) RFC Number: (P-10) ITR/Ad-hoc/47/2020-21.

## Conflict of Interest

The authors declare that the research was conducted in the absence of any commercial or financial relationships that could be construed as a potential conflict of interest.

## Publisher's Note

All claims expressed in this article are solely those of the authors and do not necessarily represent those of their affiliated organizations, or those of the publisher, the editors and the reviewers. Any product that may be evaluated in this article, or claim that may be made by its manufacturer, is not guaranteed or endorsed by the publisher.
