# Supplementary material for: Assistive Methodologies for Parkinson's Disease Tremor Management—A Health Opinion
Source: Front Public Health. 2022 Apr 26;10:850805. doi: 10.3389/fpubh.2022.850805 (PMC9087179; doi:10.3389/fpubh.2022.850805)
Supplement: Supplementary file 1 [file Presentation_1.PPTX]

## Slide 1
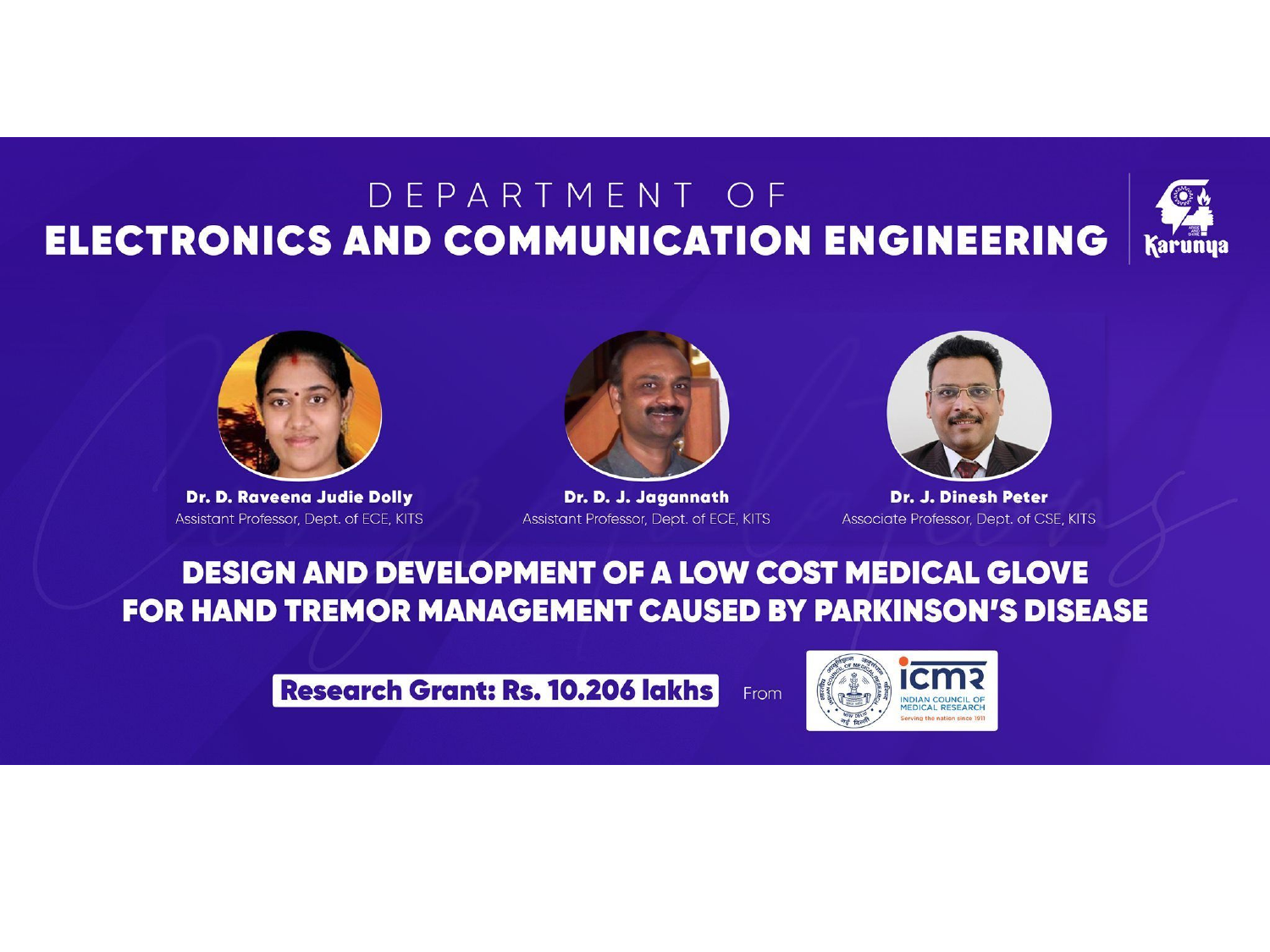

## Slide 2
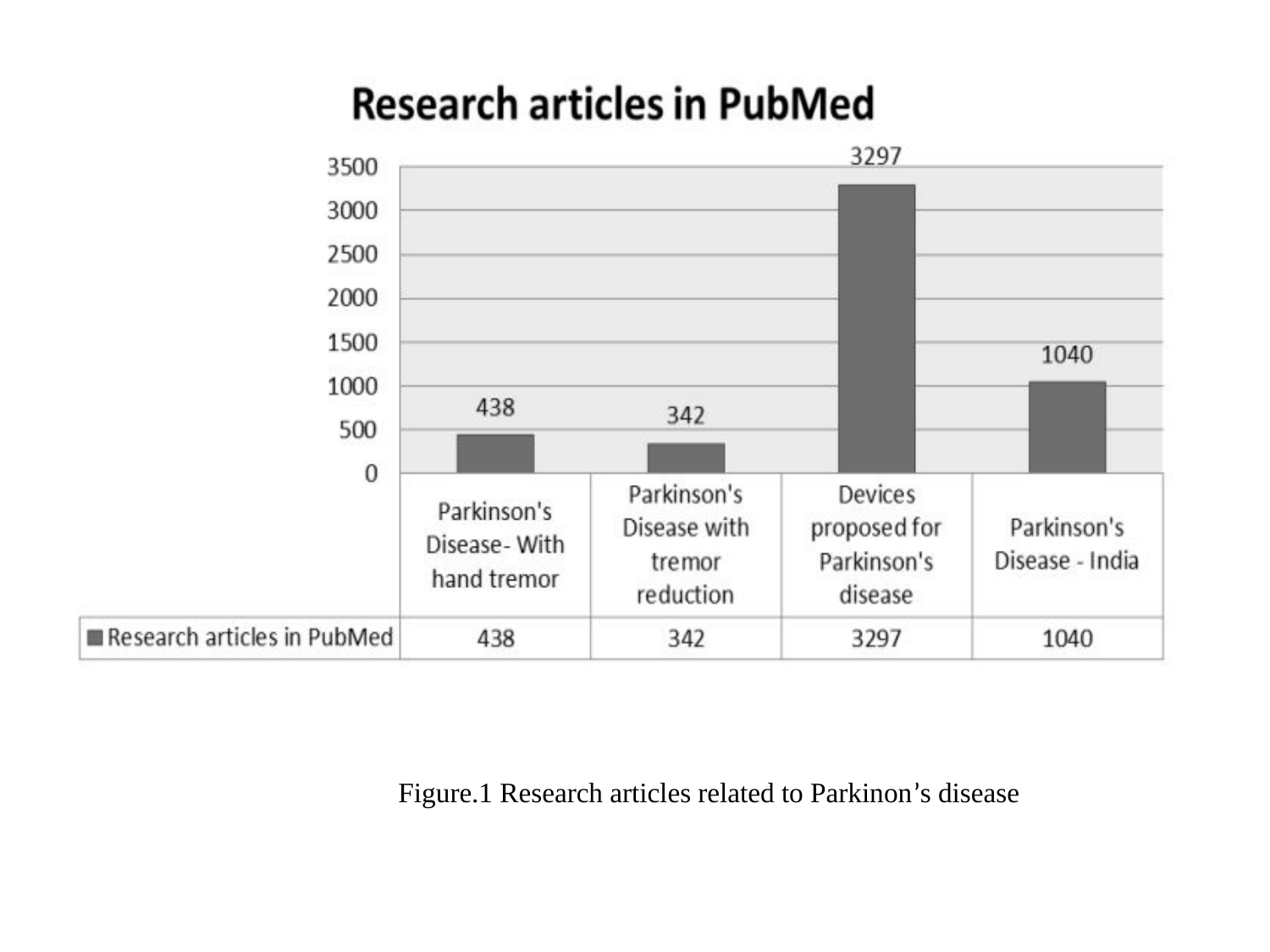

Figure.1 Research articles related to Parkinon’s disease

## Slide 3
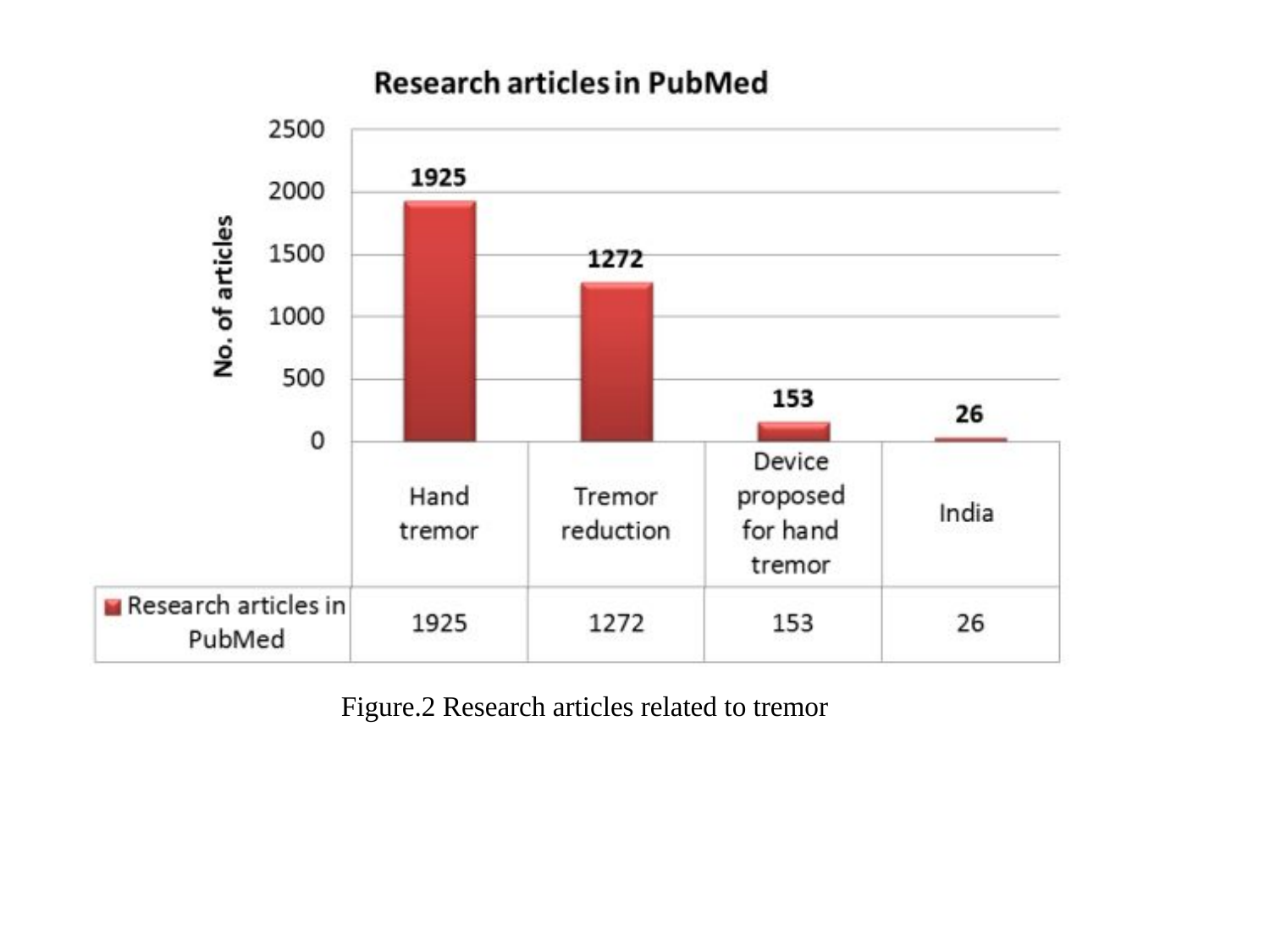

Figure.2 Research articles related to tremor

## Slide 4
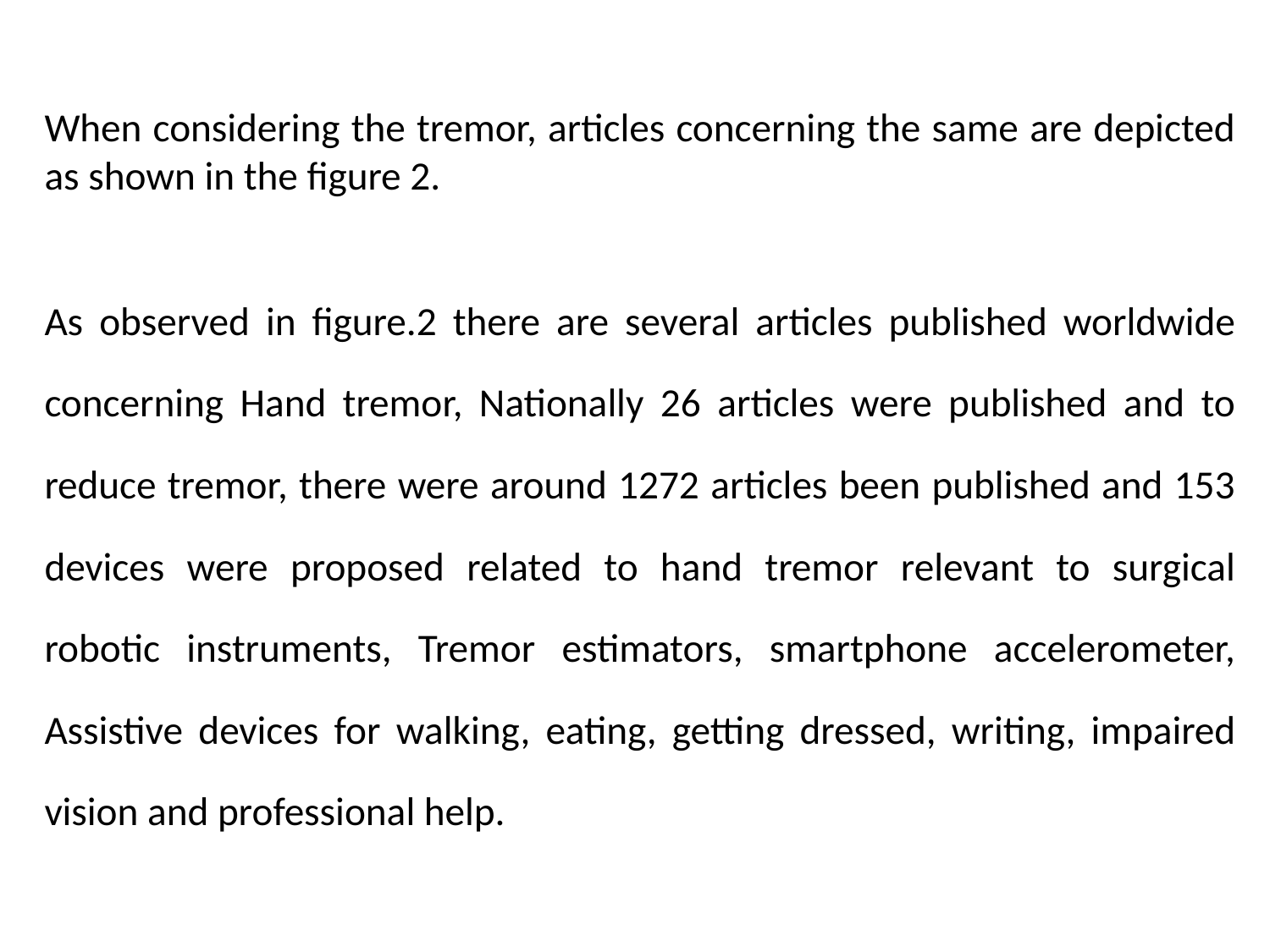

When considering the tremor, articles concerning the same are depicted as shown in the figure 2.
As observed in figure.2 there are several articles published worldwide concerning Hand tremor, Nationally 26 articles were published and to reduce tremor, there were around 1272 articles been published and 153 devices were proposed related to hand tremor relevant to surgical robotic instruments, Tremor estimators, smartphone accelerometer, Assistive devices for walking, eating, getting dressed, writing, impaired vision and professional help.
